# Supplementary material for: Social context matters: The role of social support and social norms in support for solidarity in healthcare financing
Source: PLoS One. 2023 Sep 14;18(9):e0291530. doi: 10.1371/journal.pone.0291530 (PMC10501638; doi:10.1371/journal.pone.0291530)
Supplement: S1 Table — (DOCX) [file pone.0291530.s001.docx]

**S1 Table. Spearman correlations items social support.**

Items:

1. Is there someone available to whom you can count on to listen to when you need to talk?
2. Is there someone available to you to give you good advice about a problem?
3. Is there someone available to you who shows you love and affection?
4. Is there someone available to help with daily chores?
5. Can you count on anyone to provide you with emotional support (talking over problems or helping you make a difficult decision)?
6. Do you have as much contact as you would like with someone you feel close to, someone in whom you can trust and confide in?
7. Is there someone available to you who can support you financially if needed?
8. Is there someone available to you who can help you read letters or flyers from, for example, the municipality, health insurance organisation, or hospital?
9. Are you currently married or living with a partner?

Spearman correlations between the items of the ESSI (before removal of the partner question)

|  | 1 | 2 | 3 | 4 | 5 | 6 | 7 | 8 | 9 |
| --- | --- | --- | --- | --- | --- | --- | --- | --- | --- |
| 1 | 1.0000 |  |  |  |  |  |  |  |  |
| 2 | 0.7450 | 1.0000 |  |  |  |  |  |  |  |
| 3 | 0.6059 | 0.5426 | 1.0000 |  |  |  |  |  |  |
| 4 | 0.5060 | 0.5554 | 0.5105 | 1.0000 |  |  |  |  |  |
| 5 | 0.6884 | 0.6784 | 0.6497 | 0.6110 | 1.0000 |  |  |  |  |
| 6 | 0.5952 | 0.5415 | 0.6369 | 0.5436 | 0.6846 | 1.0000 |  |  |  |
| 7 | 0.4815 | 0.5399 | 0.3952 | 0.5349 | 0.5282 | 0.4514 | 1.0000 |  |  |
| 8 | 0.5745 | 0.5574 | 0.4970 | 0.5370 | 0.5819 | 0.5055 | 0.6167 | 1.0000 |  |
| 9 | 0.1403 | 0.1296 | 0.2884 | 0.3660 | 0.2365 | 0.2443 | 0.1883 | 0.1822 | 1.0000 |

Spearman correlations between the items of the ESSI (after removal of the partner question)

|  | 1 | 2 | 3 | 4 | 5 | 6 | 7 | 8 |
| --- | --- | --- | --- | --- | --- | --- | --- | --- |
| 1 | 1.0000 |  |  |  |  |  |  |  |
| 2 | 0.7454 | 1.0000 |  |  |  |  |  |  |
| 3 | 0.6097 | 0.5454 | 1.0000 |  |  |  |  |  |
| 4 | 0.5024 | 0.5541 | 0.5072 | 1.0000 |  |  |  |  |
| 5 | 0.6894 | 0.6777 | 0.6539 | 0.6089 | 1.0000 |  |  |  |
| 6 | 0.5978 | 0.5479 | 0.6399 | 0.5430 | 0.6853 | 1.0000 |  |  |
| 7 | 0.4800 | 0.5374 | 0.3986 | 0.5364 | 0.5316 | 0.4521 | 1.0000 |  |
| 8 | 0.5670 | 0.5525 | 0.4911 | 0.5342 | 0.5795 | 0.5019 | 0.6157 | 1.0000 |
