# Supplementary material for: Social context matters: The role of social support and social norms in support for solidarity in healthcare financing
Source: PLoS One. 2023 Sep 14;18(9):e0291530. doi: 10.1371/journal.pone.0291530 (PMC10501638; doi:10.1371/journal.pone.0291530)
Supplement: S2 Table — (DOCX) [file pone.0291530.s002.docx]

**S2 Table. Results sensitivity analysis social support.**

Mean score of the scale on perceived social support for which four answers are required

| **Variable** | **N** | **Mean** | **Std. Dev.** | **Min** | **Max** |
| --- | --- | --- | --- | --- | --- |
| Perceived social support:  four answers required | 775 | 4.120 | 0.780 | 1.125 | 5 |

Mean score of the scale on perceived social support for which eight answers are required

| **Variable** | **N** | **Mean** | **Std. Dev.** | **Min** | **Max** |
| --- | --- | --- | --- | --- | --- |
| Perceived social support:  eight answers required | 757 | 4.135 | 0.766 | 1.125 | 5 |
