# Supplementary material for: Social context matters: The role of social support and social norms in support for solidarity in healthcare financing
Source: PLoS One. 2023 Sep 14;18(9):e0291530. doi: 10.1371/journal.pone.0291530 (PMC10501638; doi:10.1371/journal.pone.0291530)
Supplement: S3 Table — (DOCX) [file pone.0291530.s003.docx]

**S3 Table. Spearman correlations items social norms.**

Items:

1. My partner thinks it is important that the costs of healthcare are paid for by society as a whole. In this way, people in good health contribute to the healthcare costs of people in poor health.
2. My family thinks it is important that the costs of healthcare are paid for by society as a whole. In this way, people in good health contribute to the healthcare costs of people in poor health.
3. The people I consider important think it is important that the costs of healthcare are paid for by society as a whole. In this way, people in good health contribute to the healthcare costs of people in poor health.

Spearman correlations between the items of the social norms instrument

|  | 1 | 2 | 3 |
| --- | --- | --- | --- |
| 1 | 1.0000 |  |  |
| 2 | 0.7794 | 1.0000 |  |
| 3 | 0.7820 | 0.8268 | 1.0000 |
