# Supplementary material for: Social context matters: The role of social support and social norms in support for solidarity in healthcare financing
Source: PLoS One. 2023 Sep 14;18(9):e0291530. doi: 10.1371/journal.pone.0291530 (PMC10501638; doi:10.1371/journal.pone.0291530)
Supplement: S4 Table — (DOCX) [file pone.0291530.s004.docx]

**S4 Table. Results sensitivity analysis social norms.**

Mean score of the scale on social norms for which one answer is required

| **Variable** | **N** | **Mean** | **Std. Dev.** | **Min** | **Max** |
| --- | --- | --- | --- | --- | --- |
| Social norms:  one answer required | 758 | 3.957 | 0.889 | 1 | 5 |

Mean score of the scale on social norms for which three answers are required

| **Variable** | **N** | **Mean** | **Std. Dev.** | **Min** | **Max** |
| --- | --- | --- | --- | --- | --- |
| Social norms:  three answers required | 589 | 3.954 | 0.867 | 1 | 5 |
