# Supplementary figures and images for: Social context matters: The role of social support and social norms in support for solidarity in healthcare financing
Source: PLoS One. 2023 Sep 14;18(9):e0291530. doi: 10.1371/journal.pone.0291530 (PMC10501638; doi:10.1371/journal.pone.0291530)

**S5 Table. Logistic regression analysis – table 4, model 1.**


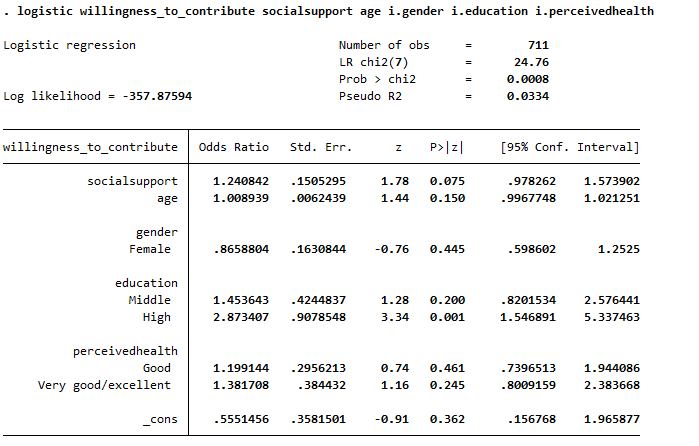

Supplement: S5 Table — (DOCX) [file pone.0291530.s005.docx]

**S6 Table. Logistic regression analysis – table 4, model 2.**


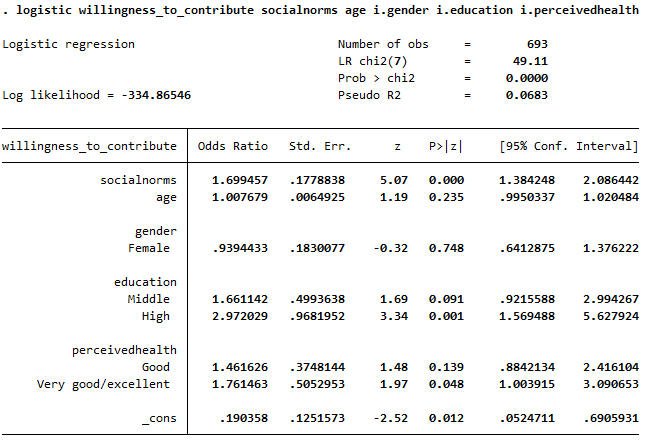

Supplement: S6 Table — (DOCX) [file pone.0291530.s006.docx]

**S7 Table. Logistic regression analysis – table 4, model 3.**


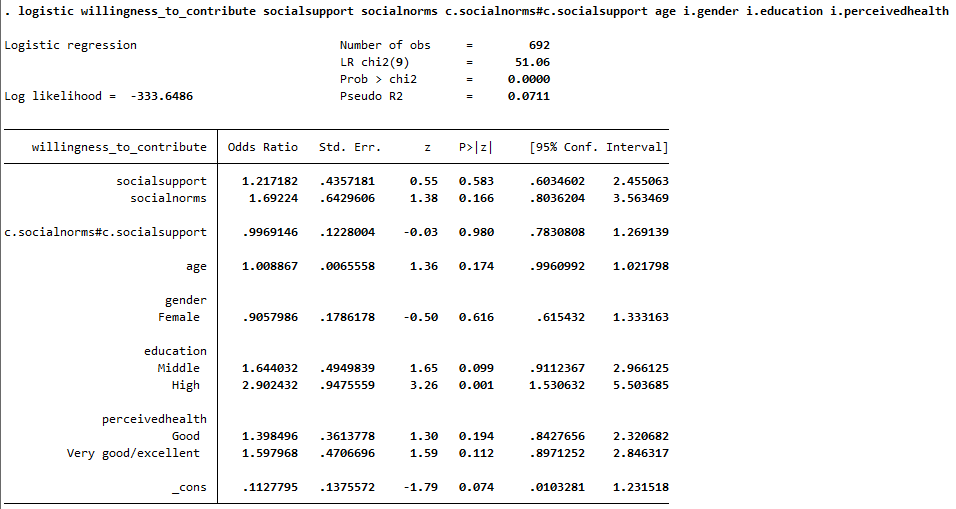

Supplement: S7 Table — (DOCX) [file pone.0291530.s007.docx]
