# Supplementary material for: Social context matters: The role of social support and social norms in support for solidarity in healthcare financing
Source: PLoS One. 2023 Sep 14;18(9):e0291530. doi: 10.1371/journal.pone.0291530 (PMC10501638; doi:10.1371/journal.pone.0291530)
Supplement: S8 Table — (DOCX) [file pone.0291530.s008.docx]

**S8 Table. Distribution of scores social support instrument.**

| **Score** | **Perceived social support in two categories** | |  |
| --- | --- | --- | --- |
|  | **Low** | **Average or high** | **Total** |
| 1.125 | 2 | 0 | 2 |
| 1.42857 | 1 | 0 | 1 |
| 1.5 | 2 | 0 | 2 |
| 1.571429 | 1 | 0 | 1 |
| 1.625 | 3 | 0 | 3 |
| 1.8 | 1 | 0 | 1 |
| 1.875 | 2 | 0 | 2 |
| 2 | 2 | 0 | 2 |
| 2.125 | 3 | 0 | 3 |
| 2.25 | 4 | 0 | 4 |
| 2.375 | 8 | 0 | 8 |
| 2.5 | 12 | 0 | 12 |
| 2.625 | 10 | 0 | 10 |
| 2.714286 | 1 | 0 | 1 |
| 2.75 | 7 | 0 | 7 |
| 2.875 | 4 | 0 | 4 |
| 3 | 24 | 0 | 24 |
| 3.125 | 13 | 0 | 13 |
| 3.25 | 13 | 0 | 13 |
| 3.333333 | 1 | 0 | 1 |
| 3.375 | 21 | 0 | 21 |
| 3.5 | 20 | 0 | 20 |
| 3.571429 | 2 | 0 | 2 |
| 3.625 | 0 | 36 | 36 |
| 3.75 | 0 | 24 | 24 |
| 3.833333 | 0 | 1 | 1 |
| 3.857143 | 0 | 2 | 2 |
| 3.875 | 0 | 37 | 37 |
| 4 | 0 | 72 | 72 |
| 4.125 | 0 | 38 | 38 |
| 4.2 | 0 | 1 | 1 |
| 4.25 | 0 | 44 | 44 |
| 4.375 | 0 | 40 | 40 |
| 4.5 | 0 | 47 | 47 |
| 4.571429 | 0 | 2 | 2 |
| 4.625 | 0 | 37 | 37 |
| 4.666667 | 0 | 1 | 1 |
| 4.75 | 0 | 66 | 66 |
| 4.857143 | 0 | 1 | 1 |
| 4.875 | 0 | 51 | 51 |
| 5 | 0 | 118 | 118 |
| **Total** | **157 (20,3%)** | **618 (79,7%)** | **775 (100%)** |
