# Supplementary material for: Social context matters: The role of social support and social norms in support for solidarity in healthcare financing
Source: PLoS One. 2023 Sep 14;18(9):e0291530. doi: 10.1371/journal.pone.0291530 (PMC10501638; doi:10.1371/journal.pone.0291530)
Supplement: Attachment — Submitted filename: Healthcare solidarity and social context_revision notes.docx [file pone.0291530.s010.docx]

**Comments from the reviewers**

**Reviewer 2**

I congratulate the authors for the updated manuscript. I agree with the findings and I accept the paper conditional on discussing the following limitations and conclusions:

Comment 1:

The possibility of non-response bias. It would be beneficial to investigate and compare the characteristics of respondents and non-respondents to assess the potential impact of non-response on the study results. Moreover, respondents with missing data on certain items were excluded, potentially leading to biased results.

Response to comment 1:

The questionnaire was sent to a sample of 1,500 panel members, representative of the Dutch adult population with regard to age and gender. The exact composition of the sample can be found below:

|  | ***Share in***  ***population*** | ***Share in sample*** | ***Men*** | ***Women*** |
| --- | --- | --- | --- | --- |
| *18-39 years old* | *34%* | *510 (34%)* |  |  |
|  |  |  | *255 (50%)* | *255 (50%)* |
| *40-64 years old* | *42%* | *630 (42%)* |  |  |
|  |  |  | *315 (50%)* | *315 (50%)* |
| *65 years and older* | *24%* | *360 (24%)* |  |  |
|  |  |  | *180 (50%)* | *180 (50%)* |
| ***Total*** | ***100%*** | ***1500*** | ***750 (50%)*** | ***750 (50%)*** |

The questionnaire was returned by 837 panel members. The composition of the group of respondents can be found in the table below:

|  | ***Share in***  ***population*** | ***Share in group of respondents*** | ***Men*** | ***Women*** |
| --- | --- | --- | --- | --- |
| *18-39 years old* | *34%* | *174 (21%)* |  |  |
|  |  |  | *65 (37%)* | *109 (63%)* |
| *40-64 years old* | *42%* | *412 (49%)* |  |  |
|  |  |  | *201 (49%)* | *211 (51%)* |
| *65 years and older* | *24%* | *251 (30%)* |  |  |
|  |  |  | *129 (51%)* | *122 (49%)* |
| ***Total*** | ***100%*** | ***837*** | ***395 (47%)*** | ***442 (53%)*** |
|  |  |  |  |  |

As can be observed from the tables above, the group of respondents consisted of fewer people in the age group 18 to 39 years (21%) compared to the general population (34%). In addition, the share of men in this age group (37%) was lower than that in the general population (50%), resulting in an underrepresentation of men in the total group of respondents (47% versus 50% in the total population). If the relationship between support for solidarity in healthcare financing and the social context among men in the age category 18 to 39 years would differ substantially from that among the rest of the population, the lower response rate in this subgroup may have led to a bias. When we looked into the relationship between support for solidarity in healthcare financing and perceived social support and social norms among men aged 18 to 39 years, we found that perceived social support is significantly associated with the willingness to contribute to other people’s healthcare costs. Men in this age category who perceive higher levels of social support are more willing to contribute to the costs of others. This is in line with the results in the total group of respondents. Contrary to the results among the total groups of respondents, we did not find a statistically significant relationship between support for solidarity in healthcare financing and social norms among men aged 18 to 39 years, although the association found was in the expected direction. Possibly, this was due to the small number of respondents in this subgroup. Therefore, we do not have a reason to assume that the relationship between support for solidarity in healthcare financing and the social context among men in the age category 18 to 39 years differs substantially from that among the rest of the population, and with that, that non-response bias has affected our results. The following text on this was included in the discussion section:

*“However, subgroup analysis showed that the relationship between support for solidarity in healthcare financing and the social context among men aged 18 to 39 years is similar to that in the rest of the population. The lower response rate in this group, therefore, does not seem to have affected our results.”*

In addition, we looked into the scores and characteristics of the respondents without missing data and the respondents with missing data and compared these two groups. This showed a similar level of support for solidarity in healthcare financing (79% versus 78%), similar mean scores on perceived social support (3.00 versus 2.96) and social norms (2.95 versus 3.02), and similar background characteristics. We added the following text about this to the strengths and limitations section:

*“In addition, we looked into any possible biases due to missing data by comparing the scores and characteristics of the respondents without missing to those of the respondents with missing data. This showed a similar level of support for solidarity in healthcare financing, similar mean scores on perceived social support and social norms, and similar background characteristics.”*

Comment 2:

Generalizability: The study was conducted in the Netherlands, and the findings may not be directly applicable to other countries or healthcare systems. Factors such as cultural norms, socioeconomic conditions, and healthcare system structures can significantly influence people's attitudes towards solidarity in healthcare financing. Therefore, caution should be exercised when generalizing the findings to different populations or countries.

Response to comment 2:

We agree with the reviewer that the specific context of the Netherlands affects people’s attitudes towards solidarity in healthcare financing. However, our study does not focus on the exact level of support for solidarity in healthcare financing in the Netherlands, but on the relationship between the degree of support and the social context. Although the level of support may vary between countries, we expect the mechanisms behind the relationship between support for solidarity in healthcare financing and the social context to be similar across countries. We added the following text about this to the strengths and limitations section:

*“Lastly, the specific context of the Netherlands, where this study was conducted, affects people's support for solidarity in healthcare financing. However, we expect the mechanisms behind the relationship between the degree of support and the social context to be similar across countries. Because of this, our findings may also be insightful for countries with a different healthcare system.”*

Comment 3:

The conclusion suggests that social support and social norms have an impact on individuals' willingness to contribute to healthcare costs. However, it is essential to consider the direction of causality. Does social support lead to a higher willingness to contribute, or do individuals with a higher willingness to contribute attract more social support?

Response to comment 3:

Since our study has a cross-sectional design, it is not possible to determine the causality between people’s support for solidarity in healthcare financing and their perceived level of social support. In our study, it is argued that people who feel more connected to others, because they perceive higher levels of social support, are more willing to contribute to the healthcare costs of others. This is done based on theories on perceived social support. However, as is stated by the reviewer, the willingness to contribute may also affect perceived social support. It is possible that the association between perceived social support and the willingness to contribute to other people’s healthcare costs works both ways and that these reinforce each other. The following text was added to the discussion section:

*“Since our study has a cross-sectional design, the causality between support for solidarity in healthcare financing and perceived social support cannot be determined. The willingness to contribute may in turn also be affected by the level of social support. Possibly, they reinforce each other.”*

Comment 4:

While the conclusion suggests that social context should be considered in healthcare financing policy, it is important to critically analyze the practical implications. How can policymakers effectively incorporate social context into policy design? What specific measures can be implemented to foster a supportive social environment for solidarity in healthcare financing?

Response to comment 4:

In accordance with the suggestion of the reviewer, we added an implications section to our paper in which we discuss the incorporation of the social context into policy design:

*“Previous studies on support for solidarity in healthcare financing have focused on mechanisms at the individual and institutional level in order to explain differences in people’s levels of support. This study gives a first insight into the role of people’s social context in their willingness to contribute to the healthcare costs of others. Our results suggest that the social context has to be taken into consideration in policy and research that addresses support for solidarity in healthcare financing. A possible way of doing this is through social norm nudges. Social norm nudges are behavioral interventions that inform individuals about the actions or attitudes of others. Since people are prone to follow social norms in order to avoid exclusion, it is expected that this information encourages them to act in a similar manner [48]. When applied to support for solidarity in healthcare financing, this could for instance be done by informing individuals about other people’s willingness to contribute to the healthcare system.”*
