# Supplementary material for: Social context matters: The role of social support and social norms in support for solidarity in healthcare financing
Source: PLoS One. 2023 Sep 14;18(9):e0291530. doi: 10.1371/journal.pone.0291530 (PMC10501638; doi:10.1371/journal.pone.0291530)
Supplement: Attachment — Submitted filename: Response to the reviewers.docx [file pone.0291530.s011.docx]

**Comments from the editor**

Comment 1

After editorial review, we notice that your supporting documents (S5-S7), pertaining to the results of the Logistic regression analysis in your manuscript, are annotated in the Dutch language. Kindly translate these Tables into English language to comply with journal requirements.

Response to comment 1

In accordance with the comment of the editor, we have translated the tables mentioned into English. Our apologies, we have overlooked that these were in Dutch.

Comment 2

In addition, kindly cross-check, and correct any other typographical or language errors within your revised manuscript.

Response to comment 2

As suggested by the editor, we checked our manuscript for any typographical or language errors and have adjusted the text where necessary.
